# Alterações Precoces nas Interleucinas Circulantes e no Risco Inflamatório Residual após Infarto Agudo do Miocárdio

**DOI:** 10.36660/abc.20190567

**Published:** 2020-12-01

**Authors:** Maria E. R. Coste, Carolina N. França, Maria Cristina Izar, Daniela Teixeira, Mayari E. Ishimura, Ieda Longo-Maugeri, Amanda S. Bacchin, Henrique Tria Bianco, Flavio T. Moreira, Ibraim Masciarelli Pinto, Gilberto Szarf, Adriano Mendes Caixeta, Otavio Berwanger, Iran Gonçalves, Francisco A. H. Fonseca

**Affiliations:** 1 Universidade Federal de São Paulo São PauloSP Brasil Universidade Federal de São Paulo, São Paulo, SP – Brasil; 2 Universidade de Santo Amaro São PauloSP Brasil Universidade de Santo Amaro, São Paulo, SP – Brasil; 3 Instituto Dante Pazzanese de Cardiologia São PauloSP Brasil Instituto Dante Pazzanese de Cardiologia, São Paulo, SP – Brasil; 4 Hospital Israelita Albert Einstein São PauloSP Brasil Hospital Israelita Albert Einstein, São Paulo, SP – Brasil

**Keywords:** Infarto do Miocárdio com Supradesnível do Segmento ST, Interleucina-6, Interleucina 10, Interleucina 18, Proteína C Reativa, Espectroscopia de Ressonância Magnética

## Abstract

**Fundamento:**

Pacientes com infarto agudo do miocárdio podem apresentar uma grande área infartada e disfunção ventricular mesmo com trombólise e revascularização precoces.

**Objetivo:**

Investigar o comportamento das citocinas circulantes em pacientes com infarto agudo do miocárdio com supradesnivelamento do segmento ST (IAMCSST) e a relação delas com a função ventricular.

**Métodos:**

No estudo BATTLE-AMI (Avaliação dos Linfócitos Tipos B e T no Infarto Agudo do Miocárdio), os pacientes com IAMCSST foram tratados com uma estratégia farmacoinvasiva. Os níveis de citocinas (IL-1β, IL-4, IL-6, IL-10 e IL-18) no plasma foram testados através de ensaio de imunoadsorção enzimática (ELISA) no início do estudo e após 30 dias. A massa infartada e a fração de ejeção ventricular esquerda (FEVE) foram examinadas por ressonância magnética cardíaca 3-T. Valores de p menores que 0,05 foram considerados significativos.

**Resultados:**

Na comparação com o início do estudo, níveis mais baixos foram detectados para IL-1β (p = 0,028) e IL-18 (p < 0,0001) após 30 dias do IAMCSST, enquanto níveis mais altos foram observados para IL-4 (p = 0,001) e IL-10 (p < 0,0001) no mesmo momento. Em contrapartida, nenhuma mudança foi detectada nos níveis de IL-6 (p = 0,63). Os níveis da proteína C-reativa de alta sensibilidade e de IL-6 se correlacionaram no início do estudo (rho = 0,45, p < 0,0001) e 30 dias após o IAMCSST (rho = 0,29, p = 0,009). No início do estudo, a correlação entre os níveis de IL-6 e FEVE também foi observada (rho = -0,50, p = 0,004).

**Conclusões:**

Durante o primeiro mês pós-infarto agudo do miocárdio, observamos uma melhora significativa no balanço das citocinas pró e anti-inflamatórias, exceto da IL-6. Esses achados sugerem risco inflamatório residual. (Arq Bras Cardiol. 2020; [online].ahead print, PP.0-0)

## Introdução

Após o infarto agudo do miocárdio (IAM), os pacientes apresentam maiores taxas de hospitalização e morte devido a insuficiência cardíaca associada a maiores níveis de proteína C-reativa de alta sensibilidade (PCRas), mas esses eventos cardiovasculares podem ser reduzidos pela terapia anti-inflamatória.^1^ Foi descrito que a interleucina-6 (IL-6) tem um papel relevante na remodelação ventricular em modelos de sobrecarga de pressão^2^ e na coordenação da resposta imune após o IAM.^3^

Além da IL-6, outras interleucinas como a IL-18^4^ e a IL-1β^5 , 6^ parecem contribuir para a remodelação ventricular adversa após o IAM. Curiosamente, a IL-4 parece contribuir para fibrose e disfunção ventricular na hipertensão arterial quando induzidas pela administração de angiotensina II.^7^ Em contrapartida, a IL-10 atenuou acentuadamente o microambiente inflamatório após o IAM, melhorando, assim, a função ventricular.^8^

Dado o interesse crescente no papel da inflamação na remodelação ventricular pós-IAM, examinamos a resposta inflamatória mediada por citocinas durante a fase precoce do IAM e a sua relação com a remodelação ventricular através de ressonância magnética cardíaca (RMC).

## Materiais e Métodos

### População do estudo

Este relato faz parte do estudo BATTLE-AMI (Avaliação dos Linfócitos Tipos B e T no Infarto Agudo do Miocárdio, ClinicalTrials.gov, NCT02428374). O BATTLE-AMI é um ensaio clínico randomizado no qual são comparados os efeitos da combinação de estatina e terapias antiplaquetárias na massa infartada e na fração de ejeção ventricular (FEVE) em pacientes com infarto agudo do miocárdio com supradesnivelamento do segmento ST (IAMCSST) tratados com uma estratégia farmacoinvasiva.^9^ Este estudo está em andamento e inclui pacientes com a primeira ocorrência de IAMCSST que foram submetidos a trombólise com tenecteplase nas primeiras 6 horas após o início dos sintomas e que foram transferidos para um hospital terciário (Hospital São Paulo) nas primeiras 24 horas para angiografia coronariana e procedimentos invasivos. Pacientes que tiveram eventos coronários prévios, revascularização da artéria coronária, contraindicações para RMC ou instabilidade hemodinâmica foram excluídos do estudo.

Este projeto foi aprovado pelo Comitê de Ética local (Universidade Federal de São Paulo, Hospital São Paulo, IRB:0297/2014, CAAE: 38692514.1.1001.5505), e todos os pacientes forneceram consentimento por escrito antes da inclusão.

### Exames laboratoriais

Amostras de sangue foram coletadas na manhã do primeiro dia e entre 27-33 dias após o IAMCSST. Todas as amostras foram testadas no Laboratório de Lípides, Aterosclerose e Biologia Vascular (Universidade Federal de São Paulo). Os níveis plasmáticos de citocinas foram testados através do ensaio imunoadsorção enzimática (ELISA). IL-4, IL-6 e IL-10 foram testadas com os kits da BD Pharmingen (BD Biosciences, San Diego, Califórnia, EUA), e IL-1β e IL-18 foram testadas com os kits da R&D (Minneapolis, Minnesota, EUA). Os resultados foram expressos em relação à absorbância usando o EnSpire Multimode Plate Reader (PerkinElmer) e/ou o iMark Microplate Absorbance Reader (Bio-Rad Laboratories, Hercules, Califórnia, EUA), de acordo com as instruções dos fabricantes. A proteína C-reativa de alta sensibilidade (PCRas) foi medida através de imunonefelometria.

### Ressonância Magnética Cardíaca

Todas as imagens de RMC foram realizadas no Hospital São Paulo ou no Instituto Dante Pazzanese de Cardiologia. A primeira avaliação foi feita nos primeiros 10 dias (início do estudo), normalmente após a alta hospitalar. A segunda avaliação foi realizada de 27 a 33 dias após o IAM.

A quantidade de massa infartada, FEVE e microcirculação foram determinadas pela RMC 3-T. Para a função ventricular esquerda, as imagens de RMC foram adquiridas através de um *scanner* 3-T, como descrito anteriormente.^9^ Em resumo, a avaliação quantitativa foi realizada em uma estação de trabalho *off-line* , com o *software* Argus LV function (Siemens Healthineers). Para a quantificação da necrose miocárdica, a planimetria foi realizada com contorno manual das áreas com realce tardio de gadolínio, e o volume do tecido infartado foi calculado como a soma dessas áreas multiplicada pela espessura de cada corte.

A cine-RMC foi realizada com uma técnica de precessão livre no estado estacionário (imagens rápidas com uso de aquisição no estado estacionário). A isquemia foi detectada através de imagens de perfusão na primeira passagem apenas na orientação do eixo curto, com pelo menos três cortes (o número máximo de cortes é limitado pela frequência cardíaca). A detecção de infarto e a quantificação das imagens foram adquiridas através da técnica de realce tardio do miocárdio após a injeção de um agente de contraste à base de gadolínio (disponível comercialmente). As imagens com meio de contraste foram adquiridas nos mesmos planos que os usados para a cine-RMC, através de uma sequência segmentada com inversão da recuperação. Cada imagem de RMC foi revisada por dois especialistas cegados e independentes usando um *software* apropriado. A função do ventrículo esquerdo foi estimada com o uso de imagens de cine-RMC para medir os volumes de FEVE e de massa de acordo com métodos padrão. As imagens com realce tardio foram utilizadas para a caracterização do infarto. Em cada paciente, após o observador ter manualmente definido a região de interesse (RDI) em um território remoto e não infartado, o tecido miocárdico foi classificado como hiper-realçado (tecido fibroso) ou miocárdio realçado normalmente.

## Análise estatística

Os dados são apresentados como média ± desvio padrão ou mediana e interquartis (IQ), de acordo com a normalidade dos dados. Variáveis contínuas foram analisadas sob o aspecto da normalidade através do teste de Kolmogorov-Smirnov. As amostras basais e de 30 dias foram comparados com o teste não paramétrico de postos de Wilcoxon. As comparações entre os grupos foram feitas através do teste de Kruskal-Wallis. Os títulos de interleucinas e os parâmetros de RMC foram correlacionados através da análise de correlação de Spearman. O tamanho amostral foi estimado com base em estudos anteriores envolvendo alterações precoces nos títulos de interleucinas.^10 , 11^ O software SPSS, versão 18.0 (IBM, Armonk, Nova Iorque, EUA), foi utilizado para a análise estatística. Valores de p < 0,05 foram considerados estatisticamente significativos.

## Resultados

### População do estudo

Um total de 139 indivíduos consecutivos com IAMCSST foram incluídos no estudo. As principais características basais da população do estudo estão descritas na Tabela 1 .

**Tabela 1:** – Características basais da população do estudo

Parâmetros	N = 139
Idade, anos*	56 (50-63)
Gênero masculino, n (%)	92 (66)
Tabagismo, n (%)	28 (20)
Diabetes, n (%)	32 (24)
HbA1c, %**	6,4 ± 1,4
Hipertensão, n (%)	82 (60)
PAS (mmHg)**	128 ± 21
PAD (mmHg)**	80 ± 14
Peso, kg**	75 ± 13
IMC, km/m^2^**	27,2 ± 4,78
Colesterol, mg/dL**	208 ± 45
Colesterol LDL, mg/dL**	138 ± 41
Colesterol HDL, mg/dL**	41 ± 12
Triglicerídios, mg/dL*	124 (86-213)
PCRas, mg/L*	15 (7-63)
Localização do infarto	
Anterior, n (%)	60 (43)
Inferior, n (%)	73 (53)
Lateral, n (%)	6 (4)

*mediana (intervalo interquartil); **média ± desvio padrão; HbA1c: hemoglobina glicada; HDL: lipoproteína de alta densidade; IMC: índice de massa corporal; LDL: lipoproteína de baixa densidade; PAD: pressão arterial diastólica; PAS: pressão arterial sistólica; PCRas: proteína C-reativa de alta sensibilidade.

### Medição das citocinas circulantes

**Figura 1:**
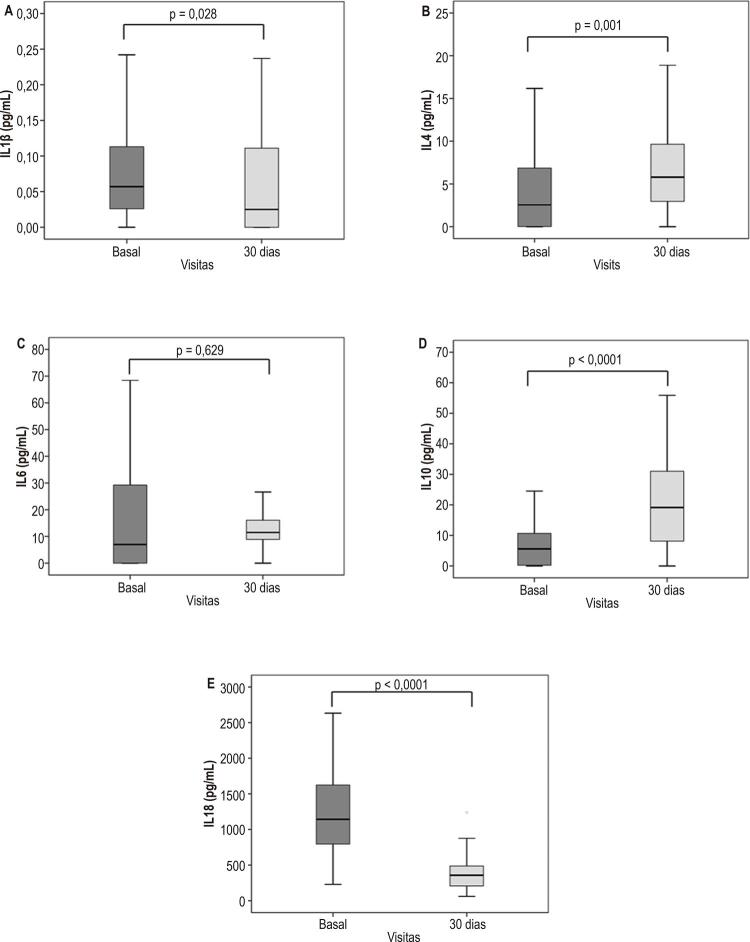
– *Gráficos em caixa das concentrações de interleucina (IL) no basal e 30 dias após o IAMCSST. (A) IL-1β; (B) IL- 4; (C) IL-6; (D) IL-10; (E) IL-18. Alterações significativas foram observadas em todas as citocinas, exceto na IL-6. Títulos foram comparados pelo teste de Wilcoxon.*

A Figura 1 mostra que, em comparação com o início do estudo, os níveis de IL-1β e IL-18 diminuíram 30 dias após o IAMCSST. Em contrapartida, foram observados aumentos nos níveis de IL-4 e IL-10 em 30 dias após o IAMCSST. Não foram observadas mudanças significativas nos níveis de IL-6 nesse período.

### Relação entre citocinas e ressonância magnética cardíaca

No início do estudo, não foram observadas correlações significativas entre os níveis de IL-1β, IL-4, IL-10 e IL-18 e os parâmetros de RMC, como a quantidade de massa infartada ou a FEVE; no entanto, houve uma correlação negativa entre os níveis de IL-6 e a FEVE (rho de Spearman = -0,50, p = 0,004). Uma tendência de correlação entre os níveis de IL-6 e a porcentagem de massa infartada do ventrículo esquerdo também foi observada (rho = 0,41, p = 0,05) ( Tabela 2 ).

**Tabela 2:** – Correlações entre interleucinas basais (pg/mL) e parâmetros de ressonância magnética cardíaca na fase aguda do infarto do miocárdio

Variáveis	rho de Spearman	Valor de p
IL-1β e massa infartada*	0,16	0,43
IL-1β e massa infartada**	-0,05	0,84
IL-1β e FEVE	0,12	0,55
IL-4 e massa infartada*	-0,26	0,19
IL-4 e massa infartada**	-0,2	0,37
IL-4 e FEVE	0,15	0,44
IL-6 e massa infartada*	0,16	0,39
IL-6 e massa infartada**	0,41	0,05
IL-6 e FEVE	-0,5	0,004
IL-10 e massa infartada*	0,3	0,1
IL-10 e massa infartada**	0,24	0,28
IL-10 e FEVE	-0,31	0,09
IL-18 e massa infartada*	-0,11	0,57
IL-18 e massa infartada**	-0,24	0,28
IL-18 e FEVE	0,01	0,96

*gramas; **porcentagem de massa ventricular esquerda; FEVE: fração de ejeção ventricular esquerda; IL: interleucina.

Houve uma correlação positiva entre os níveis basais de IL-4 e a quantidade de massa infartada medida pela RMC (rho = 0,24; p = 0,03) em 30 dias ( Tabela 3 ). Nenhuma outra correlação entre os níveis de citocinas e os parâmetros de RMC foi encontrada na avaliação de 30 dias após o IAMCSST.

**Tabela 3:** – Correlações entre concentrações basais de interleucina (pg/mL) e parâmetros de ressonância magnética cardíaca 30 dias após o infarto do miocárdio

Variáveis	rho de Spearman	Valor de p
IL-1β e massa infartada*	-0,02	0,85
IL-1β e massa infartada**	-0,07	0,59
IL-1β e FEVE	0,19	0,1
IL-4 e massa infartada*	0,24	0,03
IL-4 e massa infartada**	0,14	0,2
IL-4 e FEVE	-0,14	0,19
IL-6 e massa infartada*	0,13	0,23
IL-6 e massa infartada**	0,13	0,22
IL-6 e FEVE	-0,17	0,1
IL-10 e massa infartada*	0,13	0,23
IL-10 e massa infartada**	0,07	0,55
IL-10 e FEVE	0,09	0,4
IL-18 e massa infartada*	0,14	0,31
IL-18 e massa infartada**	-0,12	0,38
IL-18 e FEVE	0,07	0,52

*gramas; **porcentagem de massa ventricular esquerda; FEVE: fração de ejeção ventricular esquerda; IL: interleucina.

### Relação entre citocinas e proteína C-reativa de alta sensibilidade

Os níveis de PCRas se correlacionaram com os de IL-6 no início do estudo (rho = 0,45, p < 0,0001) e 30 dias após o IAMCSST (rho = 0,29, p = 0,009). Nenhuma outra citocina mostrou correlação com os níveis de PCRas tanto no início do estudo quanto 30 dias após o IAMCSST (dados não apresentados).

### Relação entre IL-6 e artéria coronária culpada

A artéria coronária direita foi a artéria coronária mais comumente culpada pelo IAMCSST (46%), seguida pela artéria descendente anterior esquerda (42%) e pela artéria circunflexa esquerda (12%). Os níveis de IL-6 constatados no primeiro dia após o IAMCSST não foram diferentes entre as artérias culpadas (p = 0,063 no teste de Kruskal-Wallis), assim como 30 dias após o IAMCSST (p = 0,131 no teste de Kruskal-Wallis).

### Ressonância magnética cardíaca

A Tabela 4 mostra os resultados da RMC de acordo com a artéria coronária culpada. Não foram observadas diferenças significativas para massa infartada (%), massa ventricular esquerda ou FEVE no início do estudo ou 30 dias após o IAMCSST.

**Tabela 4:** – Resultados da ressonância magnética cardíaca por artéria coronária culpada no início do estudo e 30 dias após infarto agudo do miocárdio

Artéria coronária culpada	Início	30 dias
Artéria descendente anterior esquerda		
Tamanho do infarto, % VE	10,0 (5,5-19,0)	12,7 (8,0-21,0)
Massa do VE, gramas	117,3 (101,0-171,8)	90,5 (69,0-127,9)
FEVE, %	46,0 (43,3-59,0)	51,6 (40,5-59,3)
Artéria coronária direita		
Tamanho do infarto, % VE	12,0 (10,0-18,5)	10,0 (6,0-17,9)
Massa do VE, gramas	96,0 (86,5-123,0)	99,0 (80,0-113,0)
FEVE, %	48,0 (42,0-50,5)	55,5 (50,0-60,0)
Artéria circunflexa esquerda		
Tamanho do infarto, % VE	11,5 (5,0-18,0)	7,0 (4,0-8,7)
Massa do VE, gramas	103,0 (76,0-130,0)	103,0 (75,0-106)
FEVE, %	51,0 (50,0-52,0)	54,0 (51,0-58,0)

*Dados apresentados como mediana (intervalo interquartil). FEVE: fração de ejeção ventricular esquerda; VE: ventrículo esquerdo. As imagens basais de ressonância magnética cardíaca foram obtidas em até 10 dias após o infarto do miocárdio. No início do estudo, nenhuma diferença foi observada no tamanho do infarto entre as artérias coronárias culpadas (p = 0,59), assim como na massa do VE (p = 0,08) ou na FEVE (p = 0,62) (teste de Kruskal-Wallis para todas as análises). Também não foram observadas diferenças entre as artérias coronárias culpadas depois de 30 dias no tamanho do infarto (p = 0,13), na massa do VE (p = 0,86) ou na FEVE (p = 0,10) (o teste de Kruskal-Wallis foi usado nessas comparações).*

## Discussão

Nosso estudo mostra o comportamento das concentrações de citocinas na fase precoce do IAMCSST em pacientes que foram tratados com uma estratégia farmacoinvasiva e receberam cuidado médico padrão. Nossos principais achados foram uma diminuição significativa nos títulos de citocinas pró-inflamatórias (IL-1β e IL-18), mas não nos de IL-6, e um aumento nos títulos de citocinas protetoras (IL-10 e IL-4). Curiosamente, a FEVE obtida pela RMC mostrou correlação com as concentrações basais de IL-6, mas não 30 dias após o IAMCSST. Além disso, a massa infartada quantificada pela RMC no período de 30 dias mostrou correlação com os níveis basais de IL-4. Em conjunto, esses dados apontam para um papel importante do perfil das interleucinas no primeiro dia após o IAM, que parece apresentar relação com a massa infartada e a remodelação ventricular. Além disso, a diminuição substancial de algumas citocinas inflamatórias juntamente com o aumento significativo da IL-10 parece atenuar, ao menos em parte, os efeitos prejudiciais da IL-6 na remodelação ventricular.^12^

A recanalização coronária precoce e o uso tanto de fármacos antitrombóticos quanto de hipolipemiantes altamente efetivos são estratégias bem estabelecidas no tratamento de pacientes com IAM. No entanto, um melhor conhecimento do risco inflamatório residual durante o acompanhamento inicial de IAM pode contribuir para novas oportunidades terapêuticas.^13^

No nosso estudo, as concentrações de IL-6 foram inversamente associadas à função ventricular esquerda. Estudos de randomização mendeliana sugeriram um papel causal da IL-6 na coronariopatia^14 , 15^ e no desenvolvimento de aneurisma de aorta abdominal.^16^ Dois grandes estudos prospectivos recentes envolvendo indivíduos após eventos coronários agudos mostraram uma associação independente entre maiores concentrações de IL-6 e desfechos cardiovasculares principais, incluindo morte cardiovascular, mesmo após múltiplos ajustes para os biomarcadores clássicos de doença cardiovascular.^17 , 18^ A interação da IL-6 com seu receptor parece modular o microambiente inflamatório em doenças cardiovasculares tanto em relação à desestabilização da placa quanto ao prognóstico de longo prazo.

Esse microambiente inflamatório, que envolve os biomarcadores endoteliais e inflamatórios, pode ser modulado pela terapia antiplaquetária escolhida.^19 , 20^ No entanto, no estudo DISPERSE-2 (Estudo de Confirmação de dose para Avaliação dos Efeitos Antiplaquetários de AZD6140 vs. Clopidogrel no IAMSSST 2),^21^ ao comparar o uso de ticagrelor com clopidogrel após síndrome coronariana aguda recente, não foram encontradas diferenças nos biomarcadores inflamatórios no início do estudo, na alta hospitalar e após 4 semanas. É possível que a diminuição acentuada nos níveis de outros marcadores inflamatórios, como IL-1β e IL-18, e o aumento nos níveis de IL-10 protetora tenham contribuído para uma resposta inflamatória mais favorável, apesar dos níveis persistentemente elevados de IL-6. Além disso, esses pacientes receberam tratamento hipolipemiante efetivo de ação anti-inflamatória comprovada, com rosuvastatina^22^ ou com uma combinação de sinvastatina e ezetimiba.^23^ Curiosamente, apenas o nível de IL-6 não se alterou após o tratamento médico, o que sugere que o controle dessa citocina requer terapia adicional, como o uso de anticorpo monoclonal ou de um fármaco que reduza a atividade inflamatória desencadeada pela IL-6.^24 , 25^

A IL-4 tem diversas propriedades biológicas, incluindo a diferenciação dos linfócitos Th1 em células com atividade inflamatória menor (Th2).^25^ Além disso, foi relatado que a IL-4 cronicamente elevada tem uma relação causal com a fibrose cardíaca e a remodelação cardíaca adversa.^26^ Além disso, a cardiomiopatia dilatada induzida pela angiotensina II é modulada pelos níveis de IL-4.^27^ Neste estudo, observamos uma associação entre os níveis basais de IL-4 e a quantidade de massa infartada depois de 30 dias do IAM. Esses achados sugerem um papel importante dessa citocina, que possivelmente atenua o processo inflamatório miocárdico através de maior diferenciação celular em fenótipos menos inflamatórios (macrófagos M2 e linfócitos Th2). Nesse cenário, a IL-4 pode influenciar todo o processo de remodelação ventricular. Este pode levar diversas semanas para ocorrer e parece depender da relação entre células inflamatórias e cardiomiócitos, determinando, assim, a eliminação de células necróticas e promovendo reposição celular e formação de tecido fibroso.^28^

Os inflamassomas são uma família do sistema imune inato que inclui o NLRP3, que foi reconhecido como um gatilho relevante para o efeito inflamatório em cascata relacionado à doença cardiovascular.^29^ Essa plataforma pode ser ativada por diversos estímulos, incluindo a hipóxia, promovendo a liberação das citocinas altamente inflamatórias IL-1β e IL-18.^30^ Além disso, a síndrome metabólica e o diabetes estão relacionados às concentrações de IL-18. Enquanto a IL-1β está relacionada ao efeito inflamatório em cascata da doença cardiovascular, a IL-18 parece estar associada a mecanismos inflamatórios, favorecendo o desenvolvimento de câncer e apresentando maiores concentrações em pacientes com diabetes e resistência à insulina.^31 , 32^ Nosso estudo mostrou uma diminuição nas duas citocinas (IL-1β e IL-18), o que sugere uma diminuição nos estímulos para ativação do NLRP3 após 30 dias do IAMCSST.

Este estudo também reforça a importância do papel da IL-6, a única citocina não modificada após 30 dias do IAM, que apresentou uma correlação significativa com a PCRas basal e 30 dias após o IAMCSST, uma associação previamente relatada.^33^ No nosso estudo, apenas pacientes com IAMCSST submetidos a trombólise nas primeiras 6 horas e encaminhados para angiografia coronariana nas primeiras 24 horas foram incluídos. Assim, esta é uma população altamente homogênea que recebeu cuidado médico padrão. Levando em consideração a associação de níveis cronicamente elevados de IL-6 e a recorrência de eventos coronários, insuficiência cardíaca, mortalidade cardiovascular e mortalidade por todas as causas, uma diminuição adicional no risco inflamatório residual parece ser um alvo promissor para a intervenção.^34 , 35^

### Limitações do estudo

A população estudada recebeu terapia hipolipemiante e antiplaquetária, cujos efeitos anti-inflamatórios podem ter contribuído para os resultados do estudo. No entanto, esses tratamentos são parte do cuidado padrão desses pacientes. Algumas citocinas inflamatórias capazes de ativar a via inflamatória mediada pela IL-6, como o fator de necrose tumoral-alfa (TNF-α) ou o IL-1R, não foram medidas e podem ter relevância nas respostas teciduais e na remodelação ventricular do paciente.^36 , 37^ Na verdade, o IAM *per se* pode estar relacionado a um aumento na IL-6 como resposta a uma lesão. No entanto, os títulos de IL-6 permaneceram elevados enquanto outras citocinas alteraram seus níveis séricos após 30 dias do IAMCSST. Outro biomarcador inflamatório importante que não foi avaliado neste estudo é a IL-1α, que é liberada pelos cardiomiócitos necróticos e ativa as respostas imunes dos fibroblastos.^38^ O bloqueio da IL-1α diminui a atividade quimiotática para diversas células mediadas por CCL2/ MCP-1 e IL-6.^38^ Além disso, o recrutamento de monócitos e linfócitos na isquemia cardíaca pode ser estimulado por diversos quimiotáticos, como CCL2 e CCL5, influenciando, assim, a cura tecidual.^39^ Por fim, o fator de transformação do crescimento beta (TGF-β), que é altamente expressado após o IAM, também não foi avaliado pelo nosso estudo, mas foi implicado na sobrevida de cardiomiócitos e na remodelação ventricular.^40^ Os resultados apresentados aqui referem-se a um período relativamente precoce após o IAM, mas é quando os infiltrados inflamatórios parecem mais relevantes para a recuperação celular ou lesão de reperfusão.

## Conclusões

Durante o primeiro mês pós-IAM, observamos uma melhora significativa no balanço das citocinas pró e anti-inflamatórias, exceto da IL-6. Esses achados sugerem risco inflamatório residual.

### Destaques

**Table:** 

As estratégias atuais de cuidado de pacientes com infarto agudo do miocárdio parecem insuficientes para modificar a via inflamatória mediada pela interleucina-6. Maiores concentrações dessa citocina parecem estar associadas a menor fração de ejeção ventricular. As terapias direcionadas à interleucina-6 parecem promissoras para diminuição adicional do risco inflamatório residual em sujeitos com infarto agudo do miocárdio. O grande desafio de reduzir o risco inflamatório residual está em desenvolver terapias seguras e acessíveis.
