# Supplementary material for: Alterações Precoces nas Interleucinas Circulantes e no Risco Inflamatório Residual após Infarto Agudo do Miocárdio
Source: Arq Bras Cardiol. 2020 Dec 1;115(6):1104–11. [Article in Portuguese] doi: 10.36660/abc.20190567 (PMC8133737; doi:10.36660/abc.20190567)
Supplement: Translation

# Translation: Early Changes in Circulating Interleukins and Residual Inflammatory Risk After Acute Myocardial Infarction

**Authors:** Maria E. R. Coste, Carolina N. França, Maria Cristina Izar, Daniela Teixeira, Mayari E. Ishimura, Ieda Longo-Maugeri, Amanda S. Bacchin, Henrique Tria Bianco, Flavio T. Moreira, Ibraim Masciarelli Pinto, Gilberto Szarf, Adriano Mendes Caixeta, Otavio Berwanger, Iran Gonçalves, Francisco A. H. Fonseca

**Affiliations:** 1 Brazil Universidade Federal de São Paulo, São Paulo, SP – Brazil; 2 Brazil Universidade de Santo Amaro, São Paulo, SP – Brazil; 3 Brazil Instituto Dante Pazzanese de Cardiologia, São Paulo, SP – Brazil; 4 Brazil Hospital Israelita Albert Einstein, São Paulo, SP – Brazil

**Keywords:** ST Elevation Myocardial Infarction, Interleukin-6, Interleukin-10, Interleukin-18, C-reactive Protein, Magnetic Resonance Spectroscopy

## Abstract

**Background:**

Patients with acute myocardial infarction may have a large infarcted area and ventricular dysfunction despite early thrombolysis and revascularization.

**Objective:**

To investigate the behavior of circulating cytokines in patients with ST-segment elevation myocardial infarction (STEMI) and their relationship with ventricular function.

**Methods:**

In the BATTLE-AMI (B and T Types of Lymphocytes Evaluation in Acute Myocardial Infarction) trial, patients with STEMI were treated with a pharmacoinvasive strategy. The plasma levels of cytokines (IL-1 *β* , IL-4, IL-6, IL-10, and IL-18) were tested using enzyme-linked immunosorbent assay (ELISA) at baseline and after 30 days. Infarcted mass and left ventricular ejection fraction (LVEF) were examined by 3-T cardiac magnetic resonance imaging. All p-values < 0.05 were considered statistically significant.

**Results:**

Compared to baseline, lower levels were detected for IL-1 *β* (p = 0.028) and IL-18 (p < 0.0001) 30 days after STEMI, whereas higher levels were observed for IL-4 (p = 0.001) and IL-10 (p < 0.0001) at that time point. Conversely, no changes were detected for IL-6 levels (p = 0.63). The levels of high-sensitivity C-reactive protein and IL-6 correlated at baseline (rho = 0.45, p < 0.0001) and 30 days after STEMI (rho = 0.29, p = 0.009). At baseline, correlation between IL-6 levels and LVEF was also observed (rho = -0.50, p = 0.004).

**Conclusions:**

During the first month post-MI, we observed a marked improvement in the balance of pro- and anti-inflammatory cytokines, except for IL-6. These findings suggest residual inflammatory risk. (Arq Bras Cardiol. 2020; [online].ahead print, PP.0-0)

## Introduction

Following acute myocardial infarction (MI), patients are at risk of higher rates of hospitalization and death due to heart failure associated with higher levels of high-sensitivity C-reactive protein (hsCRP), but these cardiovascular events can be decreased by anti-inflammatory therapy.^1^ Interleukin-6 (IL-6) has been described as having a relevant role in ventricular remodeling in pressure overload models^2^ and orchestrating the immune response after acute MI.^3^

In addition to IL-6, other interleukins such as IL-18^4^ and IL-1β^5 , 6^ seem to contribute to adverse ventricular remodeling after MI. Interestingly, IL-4 appears to contribute to fibrosis and ventricular dysfunction in arterial hypertension when induced by angiotensin II administration.^7^ Conversely, IL-10 markedly attenuates the inflammatory microenvironment after MI, thus improving ventricular function.^8^

Given the growing interest in the role of inflammation in ventricular remodeling after acute MI, we examined cytokine-mediated inflammatory response during the early phase of acute MI and its relationship with ventricular remodeling using cardiac magnetic resonance imaging (cMRI).

## Materials and Methods

### Study population

This report is part of the BATTLE-AMI (B And T Types of Lymphocytes Evaluation in Acute Myocardial Infarction) trial (ClinicalTrials.gov, NCT02428374). BATTLE-AMI is a randomized trial in which the effects of combined statin and antiplatelet therapies on infarcted mass and left ventricular ejection fraction (LVEF) in patients with ST-segment elevation myocardial infarction (STEMI) treated with a pharmacoinvasive strategy are compared.^9^ The ongoing study includes patients with first STEMI who underwent thrombolysis with tenecteplase in the first 6 hours of onset of symptoms and were transferred to a tertiary care hospital in southeastern Brazil (Hospital São Paulo) in the first 24 hours for coronary angiography and invasive procedures. Patients with previous history of coronary events, coronary revascularization, contraindication to cMRI, or showing hemodynamic instability were excluded from the study.

The project was approved by the local Ethics Committee (Universidade Federal de São Paulo, Hospital São Paulo, IRB:0297/2014, CAAE: 38692514.1.1001.5505), and all patients provided written informed consent before inclusion.

### Laboratory measurements

Blood samples were collected in the morning of the first day and between 27-33 days after STEMI. All samples were assayed in the Laboratory of Lipids, Atherosclerosis, and Vascular Biology (Universidade Federal de São Paulo). Cytokine levels in plasma were tested using the enzyme-linked immunosorbent assay (ELISA). IL-4, IL-6, and IL-10 were assayed using the BD Pharmingen kits (BD Biosciences, San Diego, California, USA), and IL-1β and IL-18 were assayed using the R&D Systems kits (Minneapolis, Minnesota, USA). The results were expressed in terms of absorbance using the EnSpire Multimode Plate Reader (PerkinElmer) and/or the iMark Microplate Absorbance Reader (Bio-Rad Laboratories, Hercules, California, USA), according to the manufacturers’ instructions. HsCRP was measured by immunonephelometry.

### Cardiac magnetic resonance imaging

All cMRI examinations were performed at Hospital São Paulo or Instituto Dante Pazzanese de Cardiologia. The first examination was done within the first 10 days (baseline), usually after hospital discharge. The second examination was performed after 27-33 days of acute MI.

The amount of infarcted mass, LVEF, and microcirculation were determined by 3-T cMRI. For left ventricular function, cMRI images were acquired using a 3-T scanner, as previously reported.^9^ Briefly, quantitative assessment was performed in an offline workstation with the software Argus LV function (Siemens Healthineers). For quantification of myocardial necrosis, planimetry was performed manually by contouring late gadolinium enhancement areas, and infarcted tissue volume was calculated as the sum of those areas multiplied by the thickness of each slice.

Cine cMRI was performed using a steady-state free-precession technique (fast imaging employing steady-state acquisition). Ischemia was detected using first-pass perfusion imaging only in the short-axis orientation, with at least three slices (the maximum number of slices are limited by heart rate). Infarct detection and quantification images were acquired using the myocardial delayed enhancement technique after injection of a commercially available gadolinium-based contrast agent. Contrast-enhanced images were acquired in the same views as those used for cine cMRI, using a segmented inversion-recovery sequence. Each cMRI image was reviewed by two independent blinded readers using a dedicated software. Left ventricular function was estimated using cine cMRI images to measure LVEF volumes and mass according to standard methods. Delayed enhancement images were used for infarct characterization. In each patient, myocardial tissue was classified as hyperenhanced (scar tissue) or normally enhanced myocardium after the observer, who used manual interaction, defined a region of interest (ROI) within a remote non-infarcted territory.

## Statistical analysis

Data are presented as mean ± standard deviation or median (interquartile range, IQR) according to data normality. Continuous variables were analyzed for normality using the Kolmogorov-Smirnov test. Baseline and 30-day samples were compared using the nonparametric Wilcoxon signed-rank test. Comparisons between groups were made using the Kruskal-Wallis test. Interleukin titers and cMRI parameters were correlated using the Spearman’s rank correlation analysis calculator. Sample size was estimated based on previous studies involving early changes in cytokine titers.^10 , 11^ The software SPSS, version 18.0 (IBM, Armonk, New York, USA), was used for statistical analysis. All p-values < 0.05 were considered statistically significant.

## Results

### Study population

In total, 139 consecutive individuals with STEMI were included in the study. The main characteristics of the study population at baseline are described in Table 1 .

**Table 1:** – Baseline characteristics of the study population

Parameters	N = 139
Age, years*	56 (50-63)
Male gender, n (%)	92 (66)
Smoking, n (%)	28 (20)
Diabetes, n (%)	32 (24)
HbA1c, %**	6.4 ± 1.4
Hypertension, n (%)	82 (60)
SBP, mm Hg**	128 ± 21
DBP, mm Hg**	80 ± 14
Weight, kg**	75 ± 13
BMI, kg/m2**	27.2 ± 4.78
Cholesterol, mg/dL**	208 ± 45
LDL-C, mg/dL**	138 ± 41
HDL-C, mg/dL**	41 ± 12
Triglycerides, mg/dL*	124 (86-213)
hsCRP, mg/L*	15 (7-63)
Myocardial infarction location	
Anterior, n (%)	60 (43)
Inferior, n (%)	73 (53)
Lateral, n (%)	6 (4)

*median (interquartile range); **mean ± standard deviation; BMI: body mass index; DBP: diastolic blood pressure; HbA1c: glycated hemoglobin; HDL-C: high-density lipoprotein cholesterol; hsCRP: high-sensitivity C-reactive protein; LDL-C: low-density lipoprotein cholesterol; SBP: systolic blood pressure.

### Measurement of circulating cytokines

Figure 1 shows that, compared to baseline, levels of IL-1β and IL-18 decreased 30 days after STEMI. Conversely, increased levels of IL-4 and IL-10 were observed 30 days after STEMI. No significant changes were observed for IL-6 levels over time.

**Figure 1:** – *Box-plots of interleukin (IL) concentrations at baseline and 30 days after STEMI. (A) IL-1ß; (B) IL-4; (C) IL-6; (D) IL-10; (E) IL-18. Significant changes were observed in all cytokines, except for IL-6. Titers were compared using the Wilcoxon test.*

### Relationship between cytokines and cardiac magnetic resonance imaging

At baseline, no significant correlations were observed between IL-1β, IL-4, IL-10, and IL-18 levels and cMRI parameters, such as the amount of infarcted mass or LVEF; however, there was a negative correlation between IL-6 levels and LVEF (Spearman’s rho = -0.50, p = 0.004). A trend for correlation between IL-6 levels and the percentage of infarcted left ventricular mass was also noted (rho = 0.41, p = 0.05) ( Table 2 ).

**Table 2:** – Correlations between baseline interleukin concentrations (pg/mL) and cardiac magnetic resonance imaging parameters in the acute phase of myocardial infarction

Variables	Spearman’s rho	P-value
IL-1β and infarcted mass*	0.16	0.43
IL-1β and infarcted mass**	-0.05	0.84
IL-1β and LVEF	0.12	0.55
IL-4 and infarcted mass*	-0.26	0.19
IL-4 and infarcted mass**	-0.20	0.37
IL-4 and LVEF	0.15	0.44
IL-6 and infarcted mass*	0.16	0.39
IL-6 and infarcted mass**	0.41	0.05
IL-6 and LVEF	-0.50	0.004
IL-10 and infarcted mass*	0.30	0.10
IL-10 and infarcted mass**	0.24	0.28
IL-10 and LVEF	-0.31	0.09
IL-18 and infarcted mass*	-0.11	0.57
IL-18 and infarcted mass**	-0.24	0.28
IL-18 and LVEF	0.01	0.96

*grams; **percentage of left ventricular mass; IL: interleukin; LVEF: left ventricular ejection fraction.

There was a positive correlation between baseline levels of IL-4 and the amount of infarcted mass as measured by cMRI (rho = 0.24; p = 0.03) at 30 days ( Table 3 ). No other correlations between cytokine levels and cMRI parameters were found when they were assessed 30 days after STEMI.

**Table 3:** – Correlations between baseline interleukin concentrations (pg/mL) and cardiac magnetic resonance imaging parameters after 30 days of myocardial infarction

Variables	Spearman’s rho	P-value
IL-1β and infarcted mass*	-0.02	0.85
IL-1β and infarcted mass**	-0.07	0.59
IL-1β and LVEF	0.19	0.10
IL-4 and infarcted mass*	0.24	0.03
IL-4 and infarcted mass**	0.14	0.20
IL-4 and LVEF	-0.14	0.19
IL-6 and infarcted mass*	0.13	0.23
IL-6 and infarcted mass**	0.13	0.22
IL-6 and LVEF	-0.17	0.10
IL-10 and infarcted mass*	0.13	0.23
IL-10 and infarcted mass**	0.07	0.55
IL-10 and LVEF	0.09	0.40
IL-18 and infarcted mass*	0.14	0.31
IL-18 and infarcted mass**	-0.12	0.38
IL-18 and LVEF	0.07	0.52

*grams; **percentage of left ventricular mass; IL: interleukin; LVEF: left ventricular ejection fraction.

### Relationship between cytokines and high-sensitivity C-reactive protein

The levels of hsCRP correlated with those of IL-6 at baseline (rho = 0.45; p < 0.0001) and 30 days after STEMI (rho = 0.29, p = 0.009). No other cytokine showed correlation with hsCRP levels either at baseline or after 30 days of STEMI (data not shown).

### Relationship between IL-6 and culprit coronary artery

Right coronary artery was the most common culprit coronary artery related to STEMI (46%), followed by left anterior descending artery (42%) and left circumflex artery (12%). IL-6 levels examined in the first day after STEMI did not differ between the culprit arteries (p = 0.063, Kruskal-Wallis test), as well as after 30 days of STEMI (p = 0.131, Kruskal-Wallis test).

### Cardiac magnetic resonance imaging

Table 4 shows the results of cMRI according to the culprit coronary artery. No significant differences were seen for infarcted mass (%), left ventricular mass, or LVEF at baseline or 30 days after STEMI.

**Table 4:** – Cardiac magnetic resonance imaging results by culprit coronary artery at baseline and 30 days after acute myocardial infarction

Culprit coronary artery	Baseline	30 days
Left anterior descending artery		
Infarct size, % LV	10.0 (5.5-19.0)	12.7 (8.0-21.0)
LV mass, grams	117.3 (101.0-171.8)	90.5 (69.0-127.9)
LVEF, %	46.0 (43.3-59.0)	51.6 (40.5-59.3)
Right coronary artery		
Infarct size, % LV	12.0 (10.0-18.5)	10.0 (6.0-17.9)
LV mass, grams	96.0 (86.5-123.0)	99.0 (80.0-113.0)
LVEF, %	48.0 (42.0-50.5)	55.5 (50.0-60.0)
Left circumflex artery		
Infarct size, % LV	11.5 (5.0-18.0)	7.0 (4.0-8.7)
LV mass, grams	103.0 (76.0-130.0)	103.0 (75.0-106)
LVEF, %	51.0 (50.0-52.0)	54.0 (51.0-58.0)

*Data presented as median (interquartile range). LV: left ventricular; LVEF: left ventricular ejection fraction. Baseline cardiac magnetic resonance imaging was obtained within 10 days of myocardial infarction. At baseline, no differences were observed for infarct size between culprit coronary arteries (p = 0.59) as well as for LV mass (p = 0.08) or LVEF (p = 0.62) (Kruskal-Wallis test for all analyses). No differences were observed between culprit coronary arteries at 30-days for infarct size (p = 0.13), LV mass (p = 0.86), or LVEF (p = 0.10) (Kruskal-Wallis test was used for comparisons).*

## Discussion

Our study shows the behavior of cytokine concentrations in the early phase of STEMI in patients who were treated with a pharmacoinvasive strategy and received standard medical care. Our main findings were a marked decrease in the titers of proinflammatory cytokines (IL-1β and IL-18), but not IL-6, and an increase in the titers of protective cytokines (IL-10 and IL-4). Interestingly, LVEF obtained by cMRI showed a correlation with IL-6 concentrations at baseline, but not 30 days post-STEMI. In addition, the infarcted mass quantified by cMRI at 30 days showed a correlation with IL-4 levels at baseline. Together, these data suggest an important role of the interleukin profile on the first day after acute MI, which seems to have some relationship with the infarcted mass and ventricular remodeling. Moreover, the substantial decline in some inflammatory cytokines combined with the remarkable increase in IL-10 appears to attenuate, at least in part, the harmful effects of IL-6 on ventricular remodeling.^12^

Early coronary recanalization and use of both antithrombotic and highly effective lipid-lowering drugs are well-established strategies in the treatment of patients with MI. However, improved knowledge of the residual inflammatory risk during the early follow-up of MI may contribute to creating new therapeutic opportunities.^13^

In our study, IL-6 concentrations were inversely associated with left ventricular function. Mendelian randomization studies have suggested a causal role of IL-6 in coronary heart disease^14 , 15^ and in the development of abdominal aortic aneurysm.^16^ Two recent large prospective studies involving individuals after acute coronary events showed an independent association between higher concentrations of IL-6 and main cardiovascular outcomes, including cardiovascular death, even after multiple adjustments to classical biomarkers of cardiovascular disease.^17 , 18^ The interaction of IL-6 with its receptor seems to modulate the inflammatory microenvironment in cardiovascular diseases related to both plaque destabilization and long-term prognosis.

This inflammatory microenvironment involving endothelial and inflammatory biomarkers may be modulated by the antiplatelet therapy chosen.^19 , 20^ However, in the large DISPERSE-2 (Dose Confirmation Study Assessing Anti-Platelet Effects of AZD6140 vs Clopidogrel in NSTEMI 2) study,^21^ comparing ticagrelor with clopidogrel after recent acute coronary syndrome, no differences were found in inflammatory biomarkers at baseline, at discharge, and after 4 weeks. The marked decrease in the levels of other inflammatory markers, such as IL-1β and IL-18, and the increase in the levels of protective IL-10 may have contributed to a more favorable inflammatory response, despite persistently high levels of IL-6. Furthermore, these patients received an effective lipid-lowering treatment of proven anti-inflammatory action, with either rosuvastatin^22^ or simvastatin/ezetimibe combination.^23^ Interestingly, only the level of IL-6 showed no change after the medical treatment, suggesting that the control of this cytokine requires additional therapy, such as the use of a monoclonal antibody or a drug that can reduce IL-6-triggered inflammatory activity.^24 , 25^

IL-4 has several biological properties, including differentiation of Th1 lymphocytes into cells with less inflammatory activity (Th2).^25^ In addition, chronically elevated IL-4 was reported to have a causal relationship with cardiac fibrosis and adverse cardiac remodeling.^26^ Furthermore, dilated cardiomyopathy induced by angiotensin II is modulated by IL-4 levels.^27^ In our study, we observed an association between baseline IL-4 levels and the amount of infarcted mass after 30 days of MI. These findings suggest an important role for this cytokine, possibly by attenuating the myocardial inflammatory process through greater cell differentiation into less inflammatory phenotypes (M2 macrophages and Th2 lymphocytes). In this setting, IL-4 may influence the entire ventricular remodeling process. It may take several weeks to occur and seems to depend on the crosstalk between inflammatory cells and cardiomyocytes, thus determining elimination of necrotic cells and promoting cell replacement and formation of the fibrotic scar.^28^

Inflammasomes are a family of the innate immune system that includes NLRP3, which has been recognized as a relevant trigger for the inflammatory cascade related to cardiovascular disease.^29^ This platform can be activated by many stimuli, including hypoxia, promoting the release of the highly inflammatory cytokines IL-1β and IL-18.^30^ Furthermore, metabolic syndrome and diabetes are related to IL-18 concentrations. Whereas IL-1β is related to the inflammatory cascade of cardiovascular disease, IL-18 seems to be associated with inflammatory mechanisms, favoring cancer development and showing higher concentrations in subjects with diabetes and insulin resistance.^31 , 32^ Our study showed a decrease in both cytokines (IL-1ß and IL-18), suggesting a decrease in the stimuli for NLRP3 activation after 30 days of STEMI.

Our study also reinforces the important role of IL-6, the only unmodified cytokine 30 days after MI, showing a significant correlation with hsCRP at baseline and 30 days after STEMI, a previously reported association.^33^ In our study, only patients with STEMI undergoing thrombolysis in the first 6 hours and referred to coronary angiography in the first 24 hours were included. Thus, this is a highly homogeneous population receiving standard medical care. Taking into account the association of chronically elevated levels of IL-6 with the recurrence of coronary events, heart failure, cardiovascular mortality, and all-cause mortality, an additional decrease in the residual inflammatory risk seems to be a promising target for intervention.^34 , 35^

### Study limitations

The study population received lipid-lowering and antiplatelet therapies whose anti-inflammatory effects may have contributed to the study results. However, these treatments are part of the standard of care for these subjects. Some inflammatory cytokines capable of activating the inflammatory pathway mediated by IL-6, such as tumor necrosis factor-alpha (TNF-α) or IL-1R, were not measured and may have relevance in host tissue responses and ventricular remodeling.^36 , 37^ In fact, MI per se could be related to an increase in IL-6 as a response to injury. However, IL-6 titers remained elevated while other cytokines changed their serum levels after 30 days of STEMI. Another important inflammatory biomarker not evaluated in the study was IL-1α, which is released from necrotic cardiomyocytes and activates the immune responses from fibroblasts.^38^ IL-1α blockade decreases chemoattractant activity for many cells mediated by CCL2/MCP-1 and IL-6.^38^ In addition, monocyte and lymphocyte recruitment in ischemic heart can be stimulated by several chemoattractants such as CCL2 and CCL5, thus influencing tissue healing.^39^ Finally, the transforming growth factor beta (TGF-β), which is highly expressed after acute MI, was not evaluated in our study as well but has been implicated in cardiomyocyte survival and ventricular remodeling.^40^

The results presented herein refer to a relatively early period after acute MI, but this is the time when inflammatory infiltrate seems to be more relevant to cell recovery or reperfusion injury.

## Conclusions

During the first month post-MI, we observed a marked improvement in the balance of pro- and anti-inflammatory cytokines, except for IL-6. These findings suggest residual inflammatory risk.

### Highlights

**Table:** 

Current strategies in the care of patients with acute myocardial infarction seem to be insufficient to modify the inflammatory pathway mediated by interleukin-6. Higher concentrations of this cytokine appear to be associated with decreased left ventricular ejection fraction. Therapies targeting interleukin-6 seem promising for their additional decrease in residual inflammatory risk in subjects with acute myocardial infarction. The great challenge for reducing residual inflammatory risk lies in the development of safe and affordable therapies.
